# Supplementary material for: An Outcome Assessment of a Single Institution’s Longitudinal Experience with Uveal Melanoma Patients with Liver Metastasis
Source: Cancers (Basel). 2020 Jan 1;12(1):117. doi: 10.3390/cancers12010117 (PMC7016993; doi:10.3390/cancers12010117)
Supplement: Supplementary file 1 [file cancers-12-00117-s001.pdf]

Supplementary Materials: An Outcome Assessment of a Single Institution's Longitudinal Experience with Uveal Melanoma Patients with Liver Metastasis

Rino S. Seedor, David J. Eschelman, Carin F. Gonsalves, Robert D. Adamo, Marlana Orloff, Anjum Amjad, Erin Sharpe-Mills, Inna Chervoneva, Carol L. Shields, Jerry A. Shields, Michael J. Mastrangelo and Takami Sato

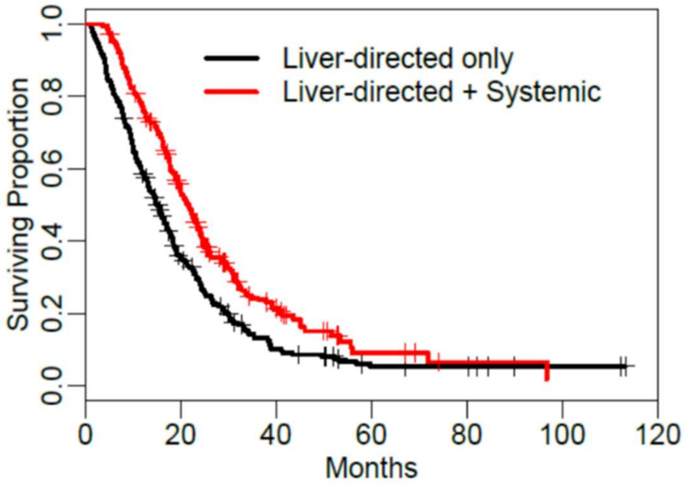

Figure S1. Effect of Systemic Treatments on Liver-directed Treatments in Cohort 3.

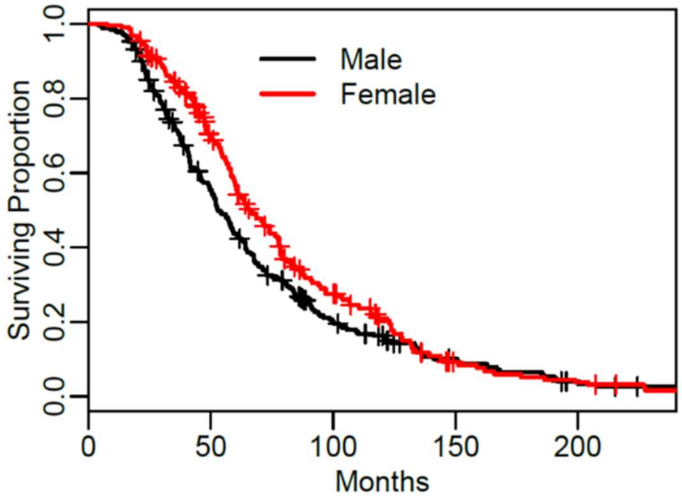

Figure S2. Effect of Gender on Eye Tx-to-Death OS in Cohort 3.

Table S1. Type of Systemic and Liver-directed Treatments (Treatment 1 + 2).

Cohort	Systemic Treatment	Chemo	Target (a)	Cytokine	Immuno (b)	Other immuno (c)	Other (d)	IE+ Target	IE+ Immuno	DEB+ Immuno	CE+ Target	CE+ Immuno	RE+ Immuno	IHA+ sutent			
**Cohort 1**	Systemic (*n* = 56)	52	0	3	0	3	1	0	0	0	0	0	0	0			
	Liver +Systemic (*n* = 8)	6	0	0	0	2	0	0	0	0	0	0	0	0			
**Cohort 2**	Systemic (*n* = 4)	5	1	1	0	0	0	0	0	0	0	0	0	0			
	Liver +Systemic (*n* = 55)	17	15	8	0	8	3	1	0	0	2	0	0	0			
**Cohort 3**	Systemic (*n* = 9)	4	6	0	7	1	0	0	0	0	0	0	0	0			
	Liver +Systemic (*n* = 192)	16	63	3	30	3	2	8	64	6	4	3	2	1			
	**Liver Treatment**	CE	IE	HP	PE	RE	DEB	Surgery	Radiation	Ablative	IE+ Target	IE+ Immuno	DEB+ Immuno	CE+ Target	CE+ Immuno	RE+ Immuno	IHA+ sutent
**Cohort 1**	Liver only (*n* = 16)	6	0	8	1	0	0	1	0	0	0	0	0	0	0	0	0
	Liver +Systemic (*n* = 8)	4	0	0	1	0	0	3	0	0	0	0	0	0	0	0	0
**Cohort 2**	Liver only (*n* = 139)	93	82	16	13	7	0	12	2	3	0	0	0	0	0	0	0
	Liver +Systemic (*n* = 55)	15	26	6	2	0	0	5	0	1	1	0	0	2	0	0	0
**Cohort 3**	Liver only (*n* = 251)	145	104	21	12	46	52	13	5	17	0	0	0	0	0	0	0
	Liver +Systemic (*n* = 192)	50	53	2	3	33	3	11	4	7	8	64	6	4	3	2	1

(a) Targeted therapy includes tyrosine kinase inhibitors, MEK inhibitors, MET inhibitors, BRAF inhibitors, BET inhibitors, PARP inhibitors, mTOR inhibitors, MDM2 inhibitors, CDK 4/6 inhibitors, PKC inhibitors, IL-1 receptor antagonists, TEM-1 antibodies, IFG-1R antibodies, valproic acid, sandostatin, hydroxychloroquine; (b) Immunotherapy includes ipilimumab, pembrolizumab, nivolumab, and tremelimumab; (c) Other immunotherapy includes IMCgp100, vaccines, Tumor-Infiltrating Lymphocytes; (d) Other includes clinical trials, intraperitoneal chemotherapy; Chemo = chemotherapy; Target = Targeted therapy; Immuno = Immunotherapy; IE = Immunoembolization; DEB = Drug eluting beads; RE = Radioembolization; CE = Chemoembolization, HP = Hepatic perfusion; PE = Plain embolization; IHA = Intrahepatic arterial infusion

Table S2. Results from the Cox Models for Overall Survival from Eye Treatment.

Cohort 1 (*N* = 80, 80 events)				
**Predictor**	**Hazard Ratio**	**LL95%CI**	**UL95%CI**	**p-value**
Age 60+ vs. < 60	1.61	1.01	2.56	0.046
**Cohort 2 (*N* = 122, 121 events, 76 patients with unknown Classification excluded)**				
**Predictor**	**Hazard Ratio**	**LL95%CI**	**UL95%CI**	**p-value**
Female vs. Male	0.59	0.41	0.87	0.007
Age 60+ vs. < 60	1.52	1.01	2.28	0.044
Ciliary vs. Choroid	1.67	1.10	2.54	0.016
**Cohort 3 (*N* = 389, 312 events, 63 patients with unknown T-stage excluded)**				
**Predictor**	**Hazard Ratio**	**LL95%CI**	**UL95%CI**	**p-value**
Age 60+ vs. < 60	1.93	1.53	2.44	<0.001
T-stage 2 vs. 1	1.32	0.91	1.91	0.145
T-stage 3 vs. 1	1.76	1.23	2.51	0.002
T-stage 4 vs. 1	2.52	1.69	3.76	<0.001
Female vs. Male				0.002(#)
(#) Supremum-test of significance of time-varying coefficient in multiplicative hazard regression model				

LL = Lower limit; CI = Confidence Interval; UL = Upper Limit

© 2020 by the authors. Licensee MDPI, Basel, Switzerland. This article is an open access article distributed under the terms and conditions of the Creative Commons Attribution (CC BY) license (http://creativecommons.org/licenses/by/4.0/).
